# Supplementary material for: A graph-based network for predicting chemical reaction pathways in solid-state materials synthesis
Source: Nat Commun. 2021 May 25;12:3097. doi: 10.1038/s41467-021-23339-x (PMC8149458; doi:10.1038/s41467-021-23339-x)
Supplement: Supplementary file 1 — Supplementary Information [file 41467_2021_23339_MOESM1_ESM.pdf]

A graph-based network for predicting chemical reaction pathways in solid-state materials synthesis

Supplementary Information

Matthew J. McDermott[1,2], Shyam S. Dwaraknath[1], and Kristin A. Persson[2,3,*]

[1]Materials Sciences Division, Lawrence Berkeley National Laboratory, 1 Cyclotron Road, Berkeley, CA 94720, USA
[2]Department of Materials Science and Engineering, University of California, Berkeley, CA 94720, USA
[3]Molecular Foundry, Lawrence Berkeley National Laboratory, 1 Cyclotron Road, Berkeley, CA 94720, USA
[*]Corresponding author: Kristin Persson (email: kapersson@lbl.gov)

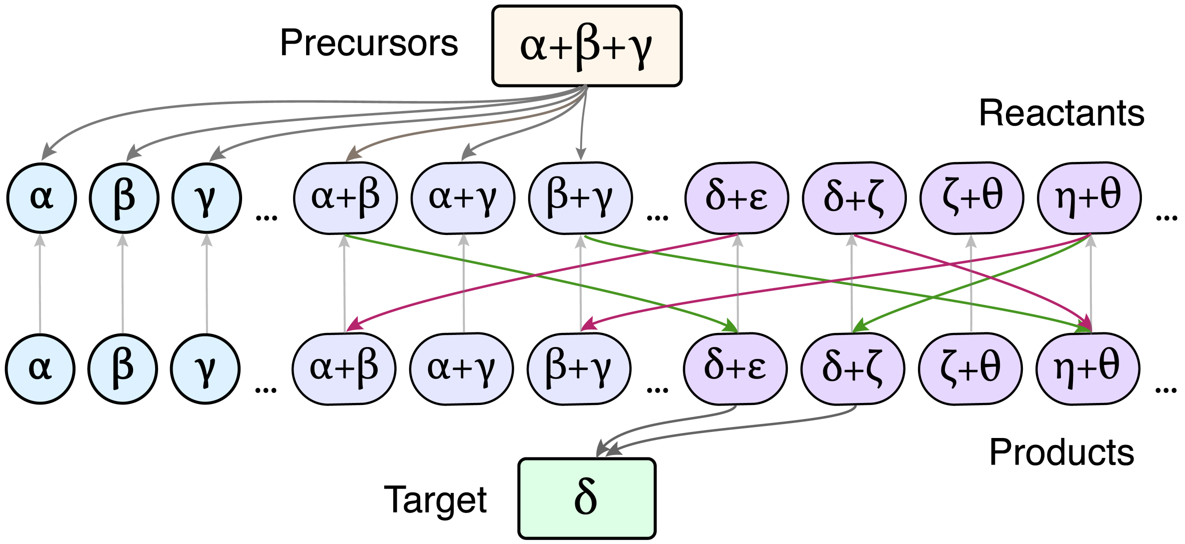

Supplementary Figure 1: Alternate schematic of reaction network illustrating reaction pathways and simple loops. The weights of edges are shaded red (high-cost), green (low-cost), and gray (zero-cost). The network depicts a hypothetical chemical system containing eight phases, labeled by Greek letters. Only two reaction pathways exist to the target, δ, starting from the precursors α, β, and γ. The first pathway is the simple one-step reaction: $\alpha + \beta \longrightarrow \delta + \epsilon$. The second pathway contains two steps: $\beta + \gamma \longrightarrow \eta + \theta \longrightarrow \delta + \zeta$. Only simple loops are illustrated here; however, the network features many more zero-cost looping edges between a product node and any reactant node which contains a subset of the product or precursor phases.
