# Supplementary material for: A graph-based network for predicting chemical reaction pathways in solid-state materials synthesis
Source: Nat Commun. 2021 May 25;12:3097. doi: 10.1038/s41467-021-23339-x (PMC8149458; doi:10.1038/s41467-021-23339-x)
Supplement: Supplementary file 3 — Description of Additional Supplementary Files [file 41467_2021_23339_MOESM3_ESM.pdf]

Description of Additional Supplementary Files

File name: Supplementary Data 1

Description: Path and entry data for the $YMnO_3$ reaction network. Includes the 76 entries in the C-Cl-Li-Mn-O-Y chemical system with predicted energies above hull < 30 meV/atom. The unfiltered list of all 38 predicted reaction pathways is shown, along with filters denoting whether the pathway has interdependent reaction steps or proceeds through hypothetical phases. Finally, each of the 20 shortest paths to $YMnO_3$, LiCl, and CO_2 are shown with their corresponding energies and costs.

File name: Supplementary Data 2

Description: Path and entry data for the $Y_2Mn_2O_7$ reaction network. Includes the 66 entries in the C-Cl-Mn-Na-O-Y chemical system with predicted energies above hull < 30 meV/atom. The unfiltered list of all 44 predicted reaction pathways is shown, along with filters denoting whether the pathway has interdependent reaction steps or proceeds through hypothetical phases. The 20 shortest paths to $Y_2Mn_2O_7$, LiCl, and CO_2 are shown with corresponding energies/costs.

File name: Supplementary Data 3

Description: Path and entry data for the Fe_2SiS_4 reaction network. Includes the 22 entries in the Fe-S-Si chemical system with predicted energies above hull < 0.5 eV/atom. The unfiltered list of all 340 predicted reaction pathways is shown, along with filters denoting whether the pathway has interdependent reaction steps or proceeds through hypothetical phases. The 75 shortest paths to Fe_2SiS_4 are shown with corresponding energies/costs.

File name: Supplementary Data 4

Description: Path and entry data for the $YBa_2Cu_3O_{6.5}$ (YBCO) reaction network. Includes the 54 entries in the Ba-Cu-O-Y chemical system with predicted energies above hull < 0.1 eV/atom. The unfiltered list of all 52 predicted reaction pathways is shown, along with filters denoting whether the pathway has interdependent reaction steps or proceeds through hypothetical phases. Finally, the 20 shortest paths to $YBa_2Cu_3O_{6.5}$ and O_2 are shown with their corresponding energies and costs.

File name: Supplementary Data 5

Description: The full 2,270 predicted paths to $MgMo_3(PO_4)_3O$ along with their energies/costs. A filter denotes whether they are considered “metathesis-like”, i.e., having a by-product that does not contain any of the target elements (Mg, Mo, P, O).

File name: Supplementary Software 1

Description: The reaction-network software (Python package) used to calculate all results in the manuscript.
