# Supplementary figures and images for: A graph-based network for predicting chemical reaction pathways in solid-state materials synthesis
Source: Nat Commun. 2021 May 25;12:3097. doi: 10.1038/s41467-021-23339-x (PMC8149458; doi:10.1038/s41467-021-23339-x)

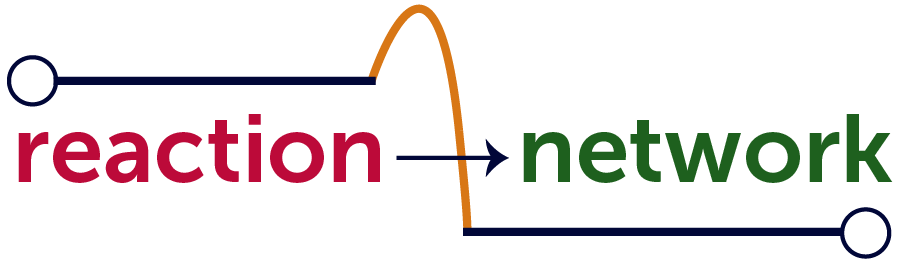

Supplement: Supplementary file 9 — Supplementary Software 1 [file 41467_2021_23339_MOESM9_ESM.zip › Supplementary Software 1/docs/images/logo.png]
